# Diabetes Adversely Influences Postoperative Outcomes After Oesophagectomy: An Analysis of the National Surgical Quality Improvement Program Database

**DOI:** 10.7759/cureus.21559

**Published:** 2022-01-24

**Authors:** Aldenb Lorenzo, David Goltsman, Christos Apostolou, Amitabha Das, Neil Merrett

**Affiliations:** 1 Upper Gastrointestinal Surgery, Bankstown-Lidcombe Hospital, Sydney, AUS; 2 General Surgery, Royal Australasian College of Surgeons, Melbourne, AUS; 3 General Surgery, Royal Australasian College of Surgeons, Sydney, AUS

**Keywords:** acs-nsqip, nsqip, surgical outcomes, wound infection, anastomotic leak, morbidity, oesophageal cancer, diabetes, complications, oesophagectomy

## Abstract

Introduction: Diabetes is a recognised risk for several chronic and acute illnesses, including increased complications in surgery for oesophageal cancer. Our primary aim is to determine the impact of diabetes on postoperative surgical and medical complications after oesophagectomy.

Methods: All oesophagectomies for malignancy as reflected in the 2016-2018 American College of Surgeons National Surgical Quality Improvement Program (ACS NSQIP) datasets were extracted and analysed. Current Procedural Terminology (CPT) codes used were 1) open procedures (43107, 43108, 43112, 43113, 43116, 43117, 43118, 43121, 43122, and 43123) and 2) hybrid procedures (43186, 43287, and 43288).

Logistic regression models examined associations between diabetic status and adverse outcomes. The associations were adjusted for sex, race, age group, operation year, CPT code, body mass index (BMI), smoking, congestive heart failure, antihypertensives, renal failure, and dyspnoea.

Results: Two thousand five hundred and thirty-eight oesophagectomies were identified. 86.45% (n=2,194) underwent open procedures and 13.55% (n=344) had hybrid procedures. There were 177 insulin-dependent diabetics (IDDM) and 320 (12.61%) non-insulin-dependent diabetics (NIDDM). 84.14% were male and 77.74% were Caucasian. 89.48% of the patients were between 50 and 79 years of age. 40.27% experienced postoperative complications. Medical complications (odds ratio [OR]: 1.7, p-value: 0.002), surgical complications (OR: 1.9, p-value: <0.001), wound complications (OR: 2.9, p-value: <0.001), and anastomotic leaks (OR: 2.4, p-value: <0.001) were more common in diabetic patients. Subgroup analysis showed that in hybrid procedures, there is a statistically significant increase in the OR of surgical complications (OR: 3.61, p-value: 0.05), medical complications (OR: 3.76, p-value: 0.04), and anastomotic leak (OR: 3.49, p-value: 0.27) in IDDM as compared to NIDDM.

Conclusion: Insulin-dependent diabetes doubles the risk of all major complications compared to nondiabetics. When considering surgical approach and diabetic status (IDDM vs nondiabetics, NIDDM vs nondiabetics), the risk of complications further doubles for hybrid oesophagectomies compared to open procedures.

## Introduction

Oesophageal cancer ranks ninth as the most common cancer and sixth as the most common cause of cancer mortality worldwide [1]. Prevalence is highest in Southern and Eastern Africa and in Eastern Asia, while lowest in Western and Central Africa and Central America in both men and women [1]. Surgery is considered the key in multimodality treatment for localised resectable oesophageal cancer. Linked with neoadjuvant therapy, surgery offers the highest likelihood of cure for patients with locoregional disease [2]. Oesophagectomy is a technically challenging operation, with a significant complication rate, magnified by patient comorbidities and anatomical challenges [3].

Oesophagectomy is associated with a relatively high surgical postoperative morbidity rate and has a high reoperation rate compared with other surgical procedures [4]. The incidence of complications related to oesophagectomy has been reported in the range of 17%-74% [5]. The 90-day perioperative mortality associated with oesophagogastric surgery has been estimated to be 5%-13% [6]. In New South Wales, Australia, the mortality related to oesophagectomy is estimated to be 4%-5% [7]. Complications associated with the surgery impact the burden of health care for patients with oesophageal surgery, including direct in-hospital costs from the length of stay, increased community nursing, family support, rehabilitation costs and costs from a delayed return to work and normal activities as well as diminished quality-of-life scores. There is evidence that complications reduce longer-term survival [8].

The increasing prevalence of diabetes was seen at the start of 2000, which parallels the increase in obesity [8]. The global incidence of diabetes increased from 108 million in 1980 to 480 million in 2014, raising the global prevalence of diabetes among adults from 4.7% (1980) to 8.5% (2014) [8]. In 2015, the economic burden caused by diabetes was US$1.31 trillion or 1.8% of global gross domestic product [9].

The increased susceptibility of diabetic patients to infection and inadequate wound healing is due to the constant stimulation of polymorphonuclear leukocytes in hyperglycaemic states making them less responsive, decreasing their capacity to fight infection [10,11]. A pervading theme of poor tissue perfusion, resulting in insufficient diffusion of antibiotics and underlying immune compromise, is considered to account for an increase in complicated skin and soft tissue infections in diabetic patients [12]. This concept has been thought to predispose to anastomotic leak and wound disruption. The increase in the levels of proinflammatory cytokines results in an insufficient immune response to pathogens and vascular inflammation, thereby contributing to poorer surgical outcomes [13].

Diabetes is considered a 21st-century epidemic that affects many chronic illnesses [13]. However, studies linking diabetes to poorer surgical outcomes, especially in oesophageal surgery, are limited [14]. No quantitative risk assessment has been conducted to stratify diabetic patients and initiate appropriate protective measures or stratify operative approaches as high or low risk for this cohort of patients. With advanced age and smoking, diabetes is associated with increased postoperative pulmonary complications in patients undergoing oesophagectomy [14]. However, several single-institution studies attempting to link diabetes to an anastomotic leak found inconsistent results owing to their small datasets [14,15].

The main objective of our study is to determine how diabetes (insulin-dependent diabetes, non-insulin-dependent diabetes, or nondiabetics) impacts postoperative outcomes dealing with surgical and medical complications in various oesophagectomy procedures using the American College of Surgeons National Quality Improvement Program (ACS-NSQIP) dataset.

This article's abstract was presented at the ANZMOSS-ANZGOSA 2020 Virtual Conference in October 2020.

## Materials and methods

ACS-NSQIP datasets

Our study evaluated over 2,500 patients to determine the association between diabetes and oesophageal surgery outcomes. The ACS-NSQIP datasets are devised of over 240 variables collected by a dedicated trained hospital staff member. For each patient included in this data set, a range of parameters are collected pertaining to their demographics, comorbidities, operation-specific information, and postoperative complications. 

Data were extracted from all oesophagectomies performed for resection of malignancy identified in the 2016-2018 ACS-NSQIP datasets. Pathology-related data for these cases had only commenced collection in 2016 as a new initiative from NSQIP collaboration. There are no totally minimally invasive oesophagectomies in the current data set analysed. There are only "hybrid" procedures where at least one body compartment is done in a minimally invasive approach. The 2018 Current Procedural Terminology (CPT) codes were used to identify these procedures. 

Dependent variables

The dependent variables examined in these analyses included anastomotic leak, wound dehiscence, deep wound infections, wound infections, reintubation, organ space infections, pulmonary embolism, pneumonia, postoperative bleed, urinary tract infection, deep venous thrombosis, sepsis, and return to the operating theatre. Three composite outcome variables were formed: major surgical complications, medical complications, and wound complications. A major surgical complication comprised a deep wound/organ space infection, a bleed requiring transfusion, or an unplanned return to the operating room within 30 days. Medical complications included any of the following: pulmonary embolism, pneumonia, postoperative renal insufficiency (creatinine > 2 mg/dL), myocardial infarction, symptomatic deep vein thrombosis, stroke, or sepsis. "Wound complications" comprised superficial surgical-site infections, deep incisional wound infections, and wound dehiscence. A separate subgroup analysis was performed to assess anastomotic leaks. 

CPT codes were subdivided into two groups: 1) open procedures (CPT codes: 43107, 43108, 43112, 43113, 43116, 43117, 43118, 43121, 43122, and 43123) and 2) hybrid procedures (CPT codes: 43186, 43287, and 43288). 

Independent variables 

In these analyses, patients were categorised into three groups: insulin-dependent diabetics (IDDM), non-insulin-dependent diabetics (NIDDM), or nondiabetics. The ACS-NSQIP criteria define a diabetic as an individual who requires exogenous parenteral insulin or oral antidiabetic agents for more than two weeks. These analyses also included patients taking antidiabetic medications to treat insulin resistance. Diet-controlled diabetics were not classified as diabetics in these analyses. 

Statistical analyses 

The baseline sociodemographic and surgical characteristics were summarised using the number and percentage in each group: IDDM, NIDDM, or nondiabetic. 

Logistic regression models examined associations between diabetic status and the various adverse outcomes. These analyses were adjusted for sex, race, age group, CPT code, body mass index (BMI), and smoking. The statistic of interest in these analyses was the odds ratios (ORs) (95% confidence interval [CI]) and their p values of the IDDM and NIDDM groups compared with the nondiabetic group. Logistic regression was also used with an interaction term for the CPT-based open and minimally invasive/hybrid groups and the outcome variables to examine if diabetic status was associated with adverse outcomes for these groups based on the surgical approach. 

## Results

Study sample

The baseline characteristics of the study sample and details of the surgeries performed are shown in Table 1. During the study period, 2,538 oesophagectomy patients were identified. The majority of these patients, 2,194 (86.45%), underwent an open procedure and 344 (13.55%) underwent minimally invasive/hybrid procedures. The cohort included 177 (6.97%) IDDM and 320 (12.61%) NIDDM. In terms of baseline demographics, 2,110 patients (84.14%) were male, and 1,973 (77.74%) were Caucasian. Age analyses showed that 89.48% of the patients were between 50 and 79 years of age at the time of surgery, 581 (22.89%) were 50-59 years old, 983 (38.78%) were 60-69 years old, and 707 (27.86%) were 70-79 years old. 

**Table 1:** Baseline characteristics IDDM, insulin-dependent diabetics; NIDDM, non-insulin-dependent diabetics; Hx, history; CCF, congestive cardiac failure; RF, renal failure; IR, interventional radiology; OR, operating room.

Variable	Statistic/level	IDDM	NIDDM	Nondiabetic	Overall
		n (% of total)	n (% of total)	n (% of total)	n (% of total)
Sex	Female	28 (1.10)	49 (1.93)	351 (13.83)	428 (16.86)
	Male	149 (5.87)	271 (10.68)	1690 (66.59)	2110 (83.14)
Race	African American	12 (0.47)	11 (0.43)	40 (1.58)	63 (2.48)
	American Indian (or other)	0 (0.00)	1 (0.04)	6 (0.24)	7 (0.28)
	Asian	11 (0.43)	14 (0.55)	59 (2.32)	84 (3.31)
	Caucasian	133 (5.24)	234 (9.22)	1606 (63.28)	1973 (77.74)
	Hawaii Indian (or other)	0 (0.00)	1 (0.04)	1 (0.04)	2 (0.08)
	Unknown	21 (0.83)	59 (2.32)	329 (12.96)	409 (16.12)
Age groups (years)	20-29	0 (0.00)	0 (0.00)	3 (0.12)	3 (0.12)
	30-39	1 (0.04)	2 (0.08)	35 (1.38)	38 (1.50)
	40-49	8 (0.32)	9 (0.35)	104 (4.10)	121 (4.77)
	50-59	32 (1.26)	59 (2.32)	490 (19.31)	581 (22.89)
	60-69	69 (2.72)	120 (4.73)	794 (31.28)	983 (38.73)
	70-79	59 (2.32)	113 (4.45)	535 (21.08)	707 (27.86)
	80-89	8 (0.32)	16 (0.63)	77 (3.03)	101 (3.98)
	>90	0 (0.00)	1 (0.04)	3 (0.12)	4 (0.16)
BMI categories	<30	85 (3.35)	185 (7.29)	1468 (57.84)	1738 (68.48)
	≥30	92 (3.62)	135 (5.32)	573 (22.58)	800 (31.52)
Smoker	Yes	32 (1.26)	66 (2.60)	527 (20.76)	625 (24.63)
	No	145 (5.71)	254 (10.01)	1514 (59.65)	1913 (75.37)
Dyspnoea	Yes	27 (1.06)	30 (1.18)	172 (6.78)	229 (9.02)
	No	150 (5.91)	290 (11.43)	1869 (73.64)	2309 (90.98)
Use of antihypertensives	Yes	48 (1.89)	3.43 (87)	1168 (46.02)	1303 (51.34)
	No	129 (5.08)	233 (9.18)	873 (34.40)	1235 (48.66)
Hx of CCF	Yes	1 (0.04)	1 (0.04)	7 (0.28)	9 (0.35)
	No	176 (6.93)	319 (12.57)	2034 (80.14)	2529 (99.65)
RF (not requiring dialysis)	Yes	0 (0.00)	1 (0.04)	1 (0.04)	2 (0.08)
	No	177 (6.97)	319 (12.57)	2040 (80.38)	2536 (99.92)
RF requiring dialysis	Yes	1 (0.04)	0 (0.00)	4 (0.16)	5 (0.20)
	No	176 (6.93)	320 (12.61)	2037 (80.26)	2533 (99.80)
Type of procedure	Open	151 (5.95)	276 (10.87)	1767 (69.62)	2194 (86.45)
	Hybrid	26 (1.02)	44 (1.73)	274 (10.80)	344 (13.55)
Medical complications	Yes	57 (2.25)	76 (2.99)	436 (17.18	569 (22.42)
	No	120 (4.73)	244 (9.61)	1605 (63.24)	1969 (77.58)
Surgical complications	Yes	73 (2.88)	95 (3.74)	526 (20.72)	694 (27.34)
	No	104 (4.10)	225 (8.87)	1515 (59.69)	1844 (72.66)
Wound complications	Yes	117 (4.61)	26 (1.02)	20 (0.72)	163 (6.42)
	No	1924 (75.81)	151 (5.95)	300 (11.82)	2375 (93.58)
Leaks	Leak, treated conservatively	10 (0.39)	12 (0.47)	69 (2.72)	91 (3.59)
	Leak treated with IR	14 (0.55)	15 (0.59)	87 (3.43)	116 (4.57)
	Leak taken back to OR	19 (0.75)	24 (0.95)	105 (4.14)	148 (5.83)
	No leak	134 (5.28)	269 (10.60)	1780 (70.13)	2183 (85.98)

Major complications and diabetic status

Overall, 40.27% (1,022) of patients experienced postoperative complications. The most common complications were classified as major surgical complications, impacting 27.34% (694) of patients in the cohort (Table 2). Logistic regression analyses showed that only IDDM had a higher likelihood of each of the major complications: medical complications (OR: 1.7, p-value: 0.002), surgical complications (OR: 1.9, p-value: <0.001), wound complications (OR: 2.9, p-value: <0.001), and anastomotic leaks (OR: 2.4, p-value: <0.001). When considering hybrid procedures, subgroup analysis demonstrated that there is a statistically significant increase in the OR of surgical complications (OR: 3.61, p-value: 0.05), medical complications (OR: 3.76, p-value: 0.04), and anastomotic leak (OR: 3.49, p-value: 0.27) in IDDM as compared to NIDDM (Table 3).

**Table 2:** Logistic regression analysis of major complication categories between diabetics and nondiabetics IDDM, insulin-dependent diabetics; NIDDM, non-insulin-dependent diabetics.

Parameter	Odds ratio		Overall p-value	
	IDDM	NIDDM	IDDM	NIDDM
Medical complications	1.7	n/a	0.002	0.6
Surgical complications	1.9	n/a	<0.001	0.32
Wound complications	2.9	n/a	<0.001	0.69
Anastomotic leak	2.4	n/a	<0.001	0.095

**Table 3:** Logistic regression analysis of the effect of surgical approach between diabetics and nondiabetics IDDM, insulin-dependent diabetics; NIDDM, non-insulin-dependent diabetics.

Parameter	Open procedures				Hybrid procedures			
	Odds ratio		p-value		Odds ratio		p-value	
	IDDM	NIDDM	IDDM	NIDDM	IDDM	NIDDM	IDDM	NIDDM
Medical complications	1.52	n/a	0.026	0.56	3.61	n/a	0.005	0.84
Surgical complications	1.82	n/a	<0.001	0.29	3.76	n/a	0.004	0.8
Wound complications	2.8	n/a	<0.001	0.82	n/a	n/a	0.65	0.73
Anastomotic leak	2.37	n/a	<0.001	0.18	3.49	n/a	0.027	0.24

## Discussion

This study aimed to objectively assess the association between diabetes mellitus and the complications in patients who underwent oesophagectomy. With the inclusion of 2,538 patients utilising the ACS NSQIP database, this is the most extensive study to date evaluating the impact of diabetes on the outcome of oesophagectomies utilising international multi-institutional data. Information on post-oesophagectomy outcomes, including anastomotic leak rates, have been included in the ACS-NSQIP since the start of 2016. There are no totally minimally invasive oesophagectomies. There are only "hybrid" procedures where at least one body compartment is accessed via a minimally invasive approach. 

Diabetes is regarded as an epidemic and is one of the biggest challenges healthcare systems face globally. The International Diabetes Federation estimated that there were 425 million people with diabetes in 2017, which is expected to increase by almost 50% in 2045 [16]. Approximately 1.3 million Australian adults (5.3% of those aged 18 and over, excluding diet-controlled and gestational diabetes) had diabetes in 2017-18, according to self-reported data from the Australian Bureau of Statistics (ABS) 2017-18 National Health Survey [17]. The age-specific rates were higher for males than females from age 45 onwards. The rate of rise in diabetes doubled among those living in the lowest socioeconomic areas (7.0%) compared with the highest socioeconomic areas (3.3%) [17]. The ABS 2018-19 National Aboriginal and Torres Strait Islander Health Survey revealed that approximately 7-8% of Indigenous and Pacific Islander populations have diabetes mellitus [18]. 

Furthermore, indigenous Australians were almost three times as likely to have diabetes as their non-indigenous counterparts [18]. Thus, determining the social factors of health is crucial in the medical care of diabetic patients. Walker et al. showed that the cultural and social background of diabetic patients largely influences blood sugar and blood pressure regulation that impacts surgical outcomes, yet little attention is placed on these factors [19]. It is important to note that cognitive-behavioural factors such as support services, stressors, and environmental factors significantly affect blood sugar control and the general well-being of patients with diabetes [19]. This makes preoperative planning on diabetic patients a complex endeavour where sociodemographic and cognitive factors are vital points to be deliberated on.

Diabetes mellitus has a significant effect on our patient cohort. Our findings indicate that the odds of an IDDM having a medical complication, surgical complication, wound complication, or anastomotic leak were, respectively, 1.7, 1.9, 2.9, and 2.4 times higher than nondiabetic patients undergoing an oesophagectomy. Our data substantiate the findings of Li et al., who, in a systematic review and meta-analysis of 12,359 patients undergoing oesophagectomy for cancer, found that diabetes mellitus is an independent risk factor for anastomotic leak and postoperative complications (OR: 1.63, 95% CI: 1.12-1.25, p < 0.01) [20]. Similarly, Booka et al. showed that in intermediate/major elective surgeries, other than cardiovascular or cerebrovascular surgery, random postoperative blood glucose increase indicates the increased incidence of poor postoperative outcomes [21]. A prospective study by Rutegard et al. showed that patients with complications after oesophagectomy had decreased survival as contrasted to those without complications (15 versus 22 months) [22]. For patients who survived more than three months, the mortality rate is higher in patients who experienced post esophagectomy complications (hazard ratio [HR] 1.29, 95% CI: 1.02-1.63) [22]. Our data offer feasible benchmarks in diabetic patients undergoing oesophageal surgery.

For all open oesophagectomies, similar findings were observed in all major complications for IDDM. Logistic regression analysis showed that for this population there was an increase in risk of medical complications (OR: 1.52, p-value: 0.026), surgical complications (OR: 1.82, p-value: <0.001), wound complications (OR: 2.80, p-value: <0.001), and anastomotic leak (OR: 2.37, p-value: <0.001) compared with a nondiabetic undergoing an oesophagectomy. 

Minimally invasive or hybrid oesophagectomy has been investigated in the past and is advantageous over open oesophagectomy [23]. However, most of the reports were retrospective, the sample size is small, and there is no consensus on which operative method is superior [24]. A recent meta-analysis comparing minimally invasive with open oesophagectomy found a decrease in morbidity with the minimally invasive approach due to the reduction of pulmonary complications, which may be explained by the absence of a thoracotomy and its associated morbidity [24]. Our study is the first to report the higher incidence of complications in diabetic patients undergoing hybrid oesophagectomies compared to open oesophagectomies.

There was only an increase in the risk of medical complications (OR: 3.61, p-value: 0.005), surgical complications (OR: 3.76, p-value: 0.004), and anastomotic leak (OR: 3.49, p-value: 0.027) in patients with IDDM compared with NIDDM when considering hybrid procedures. It is expected that there is a higher incidence of wound complications in the open versus minimally invasive procedures, as reflected in our results. Shah and Hux demonstrated that the probability of developing any infection is higher in diabetics, including postoperative infections (RR=2.02; 95% CI: 1.80-2.27), septicaemia (RR=2.45; 95% CI: 2.23-2.68), and soft tissue infections (RR=1.81; 95% CI: 1.76-1.86) [25]. This is consistent with our findings that patients with insulin-dependent diabetes are significantly linked to higher surgical, wound, and medical complications.

A contemporary meta-analysis by Yang et al. comparing minimally invasive/hybrid oesophagectomy with open oesophagectomy has demonstrated the decreased frequency of complications and in-hospital mortality occurring in minimally invasive compared with open esophagectomy, which is usually attributed to a decrease in pulmonary complications [26]. A 2019 systematic review and meta-analysis of 55 studies including over 14,000 patients demonstrates that minimally invasive and hybrid esophagectomy was associated with fewer medical and surgical complications with similar oncologic outcomes with associated 18% lower five-year all-cause mortality compared with open esophagectomy (HR: 0.82, 95% CI: 0.76-0.88) [26]. Although our study did not deal with the mortality rate associated with oesophagectomy procedures, our results show that patients with insulin-dependent diabetes undergoing hybrid procedures have a three-fold risk of medical, surgical, wound complications and anastomotic leak as compared to IDDM undergoing open oesophagectomies (Table 3).

Our study identified an objective link between diabetes and a range of postoperative complications in oesophagectomies performed for excision of malignancies using the largest cohort. However, there are several limitations of these analyses. The CPT coding system on how patient procedures were identified in the database is subject to categorisation changes on a yearly basis. These small changes in procedure codes lead to changes and variability in how a procedure may be categorised, and, in some instances, procedures become combined and cannot be included as a single entity leading to discrepancies. The big number of cases included in this study that account for these small deviations in the CPT codes over the years would likely minimally affect the results. In essence, this study has adequate power to substantiate our findings despite this potential variability. To provide a more detailed evaluation of the actual effect of blood sugar level instability, an analysis of haemoglobin A1c levels is required. Presently the ACS-NSQIP dataset does not contain this information. This dataset also does not include diet-controlled diabetics, which limits the ability to perform a more nuanced analysis. Another limitation of the ACS-NSQIP dataset is the lack of long-term follow-up, only following up patients for 30 days postoperatively. The collection of oncological data for primary malignancies has been a new initiative by the ACS-NSQIP collaborative, and this is the first study to analyse outcomes from this large cohort. More time will allow for an even higher-powered analyses and will require subsequent analyses after a substantial amount of time to allow for further maturity of the database.

Furthermore, our study does not consider the location of the anastomosis (neck vs chest), cancer site in the oesophagus, and type of anastomosis (stapled vs handsewn) used, which can all influence outcomes. Generally speaking, a cervical anastomosis is associated with a greater risk of anastomotic leak, stricture, recurrent laryngeal nerve injury, and swallowing dysfunction. An intrathoracic anastomosis can be associated with more cardiopulmonary complications and dreadful consequences in the presence of anastomosis leak [27,28]. The experience of the surgeon performing the procedure should be taken into account. A retrospective study by Tapias et al. indicated a decreased perioperative morbidity and mortality with increased surgeon experience. In this study, the learning curve for minimally invasive esophagectomy was estimated to be 25-30 procedures [29]. The follow-up period for NSQIP is restricted to 30 days postoperatively, making long-term complication data difficult to draw conclusions from. Hence, the database does not determine certain complications such as anastomotic stricture that require longer follow-up. The study also does not consider the anastomotic location, influencing the associated complications. Moreover, parameters such as the haemoglobin A1c were not available for the analysis to ascertain an understanding of patient's true glycaemic control. 

## Conclusions

This outcome-based study aims to guide clinicians with substantial risks that are easy to understand for patients with diabetes. Once a patient is an IDDM, the risk of all major complications almost doubles compared to nondiabetics. Subgroup analysis further shows that when considering the surgical approach and diabetes status, the risk of complications further doubles for minimally invasive procedures compared to open oesophagectomy (the gold standard).
